# Case studies of two related Chinese patients with Carney complex presenting with extensive cardiac myxomas and PRKAR1A gene mutation of c.491_492delTG

**DOI:** 10.1186/s12957-015-0470-4

**Published:** 2015-02-27

**Authors:** Hongwei Guo, Jianping Xu, Hui Xiong, Shengshou Hu

**Affiliations:** State Key Laboratory of Cardiovascular Disease, Fuwai Hospital, National Center for Cardiovascular Diseases, Chinese Academy of Medical Sciences and Peking Union Medical College, Beijing, 100037 China

**Keywords:** Carney complex, PRKAR1A, Mutation, Myxomas

## Abstract

Carney complex is an autosomal dominant disease that is clinically characterized by cardiac myxomas, spotty skin pigmentation, and endocrine overactivity. Carney complex is most commonly caused by mutations in the PRKAR1A gene on chromosome 17q22-24. Currently, there are at least 117 pathogenic mutations in PRKAR1A that have been identified. Herein, we report on two cases of Carney complex in related Chinese patients with a c.491_492delTG mutation that presented with multiple and extensive cardiac myxomas and skin pigmentation.

## Background

Carney complex was initially described by J. Aiden Carney in 1985. It is an autosomal dominant disorder characterized by myxomas, spotty pigmentation, and endocrine overactivity. The initial diagnosis of Carney complex is based on clinical criteria and can be confirmed using genetic testing. The genetic tests have a mutation detection rate of approximately 60% in patients with Carney complex. There are 12 clinical criteria that are the major diagnostic indicators of Carney complex. In addition, there are two supplemental diagnostic criteria that pertain to genetic testing and family history [1,2].

The genetic disease Carney complex includes two distinct syndromes: LAMB syndrome (lentigines, atrial myxomas, mucocutaneous myxomas, and blue nevi) and NAME syndrome (nevi, atrial myxoma, myxoid neurofibroma, and ephelides). These are autosomal dominant conditions that comprise myxomas in the heart and skin, skin hyperpigmentation (lentiginosis), and endocrine-related overactivity or tumors [1]. Carney complex is most commonly caused by mutations in the PRKAR1A gene on chromosome 17q22-24 [3]. It is less commonly caused by a variety of genetic changes on chromosome 2p16. Currently, 117 different PRKAR1A mutations have been identified [4-7]. Herein, we identify two Chinese Carney complex patients that are siblings with a 491_492delTG PRKAR1A gene mutation. The patients presented with multiple and extensive cardiac myxomas and skin pigmentation.

## Case presentation

### Case report 1

**Figure 1:**
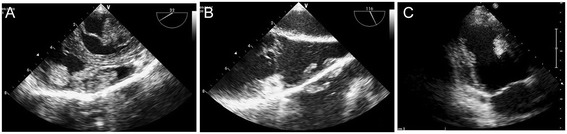
**Identification of cardiac myxomas by echocardiogram. (A)** Patient 1 - pre-operative myxomas in the left atrium and right ventricle. **(B)** Patient 1 - pre-operative myxomas in the left ventricle. **(C)** Patient 2 - post-operative recurrent myxoma in the left ventricle 3 months after the operation.

**Figure 2:**
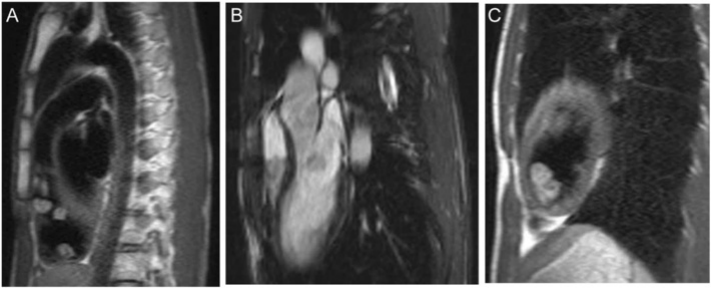
**Identification of cardiac myxomas by MRI.** The presence of cardiac myxomas was confirmed by MRI in patient 1. **(A)** Right ventricle. **(B)** Left atrium. **(C)** Left ventricle.

**Figure 3:**
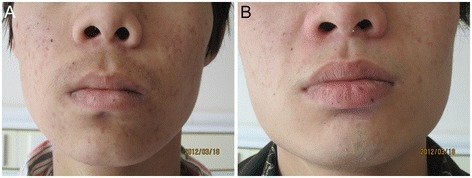
**Clinical presentation of skin hyperpigmentation.** Both patients presented with spotty skin pigmentation on their faces. **(A)** Patient 1. **(B)** Patient 2.

The first patient is a 14-year-old male who was admitted to Fuwai Hospital, Beijing, in March 2012 with intermittent syncope that had been ongoing over 2 years. Echocardiography showed multiple cardiac myxomas in the left atrium, left ventricle, and right ventricle (Figure 1A,B). The cardiac myxomas in the left atrium, left ventricle, and right ventricle were confirmed by MRI examination (Figure 2). Upon physical examination, the patient had spotty skin pigmentation on his face (Figure 3). The results of further evaluations are presented in Table 1.

**Table 1:** **Further evaluation of the two patients**

	**Case 1 (14-year-old male)**	**Case 2 (22-year-old male)**
Blood glucose	Normal	Normal
MRI pituitary examination	No sign of pituitary microadenoma was found in sellar MRI scanning	A low signal shadow with unclear boundary was found at the lower left medial side of the pituitary
CT adrenal gland examination	No obvious lesion in the bilateral adrenal glands was identified	No obvious lesion in the bilateral adrenal glands was identified
Examination of Cushing symptoms	Cortisol: 457.9 nmol/L (8 o’clock), 327.2 nmol/L (16 o’clock), 197.3 nmol/L (0 o’clock), corresponding adrenocorticotrophic hormone (ACTH) (8/16/0 o’clock): 18.57 pg/ml; 8.85 pg/ml; <1.0 pg/ml; 24-h urinary free cortisol (UFC): 309.4 nmol/24 h	Cortisol: 353.4 nmol/L(8 o’clock), 218.6 nmol/L (16 o’clock), 70.8 nmol/L (0 o’clock), adrenocorticotrophic hormone (ACTH) (8/16/0 o’clock): 41.80 pg/ml; 31.53 pg/ml; 22.73 pg/ml; 24-h urinary free cortisol (UFC): 308.4 nmol/24 h

**Figure 4:**
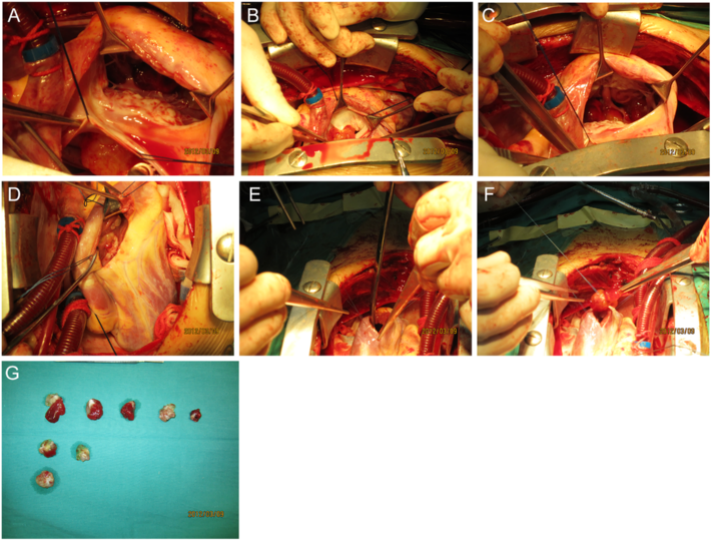
**Surgical resection of cardiac myxomas from patient 1.** Cardiac myxomas removed from patient 1 **(A)** through the interatrial septum incision; **(B)** attached to the anterior mitral valve; **(C)** through the tricuspid valve; **(D)** through an incision of right ventricular outflow tract; and **(E, F)** through a left ventricular apical incision. **(G)** After excision, the myxomas grouped by point of origin in the heart. Right ventricle (the first row); left ventricle (the second row); and left atrium (the third row).

The patient underwent surgery on 9 March 2012. The surgeon performed a median sternotomy and cardiopulmonary bypass using bicaval cannulation and ascending aorta cannulation with cardiac arrest. Myocardial protection was achieved by antegrade cardioplegia with St. Thomas solution. A 18 × 20 × 20 mm^3^ myxoma attached to the anterior mitral valve was removed through an interatrial septum incision (Figure 4A,B). Five myxomas were discovered in the right ventricle attached to cardiac muscle. Three myxomas measuring 18 × 20 × 20, 20 × 20 × 20, and 18 × 20 × 22 mm^3^ were in the inlet area or trabecular area and were removed through the tricuspid valve (Figure 4C). The remaining two myxomas measuring 6 × 8 × 8 and 8 × 8 × 8 mm^3^ were in the outlet area and were removed through a right ventricular outflow tract incision (Figure 4D). From the left ventricle, two myxomas (13 × 15 × 15 and 14 × 15 × 16 mm^3^) attached to the apical portion were removed through a left ventricular apical incision (Figure 4E,F). In total, eight myxomas were excised from the patient (Figure 4G).

During the operation, the aortic cross-clamp time was 110 min and the cardiopulmonary bypass time was 146 min. The patient was in need of assisted mechanical ventilation for 15.55 h. Patient 1 remained in the intensive care unit for 20.80 h and had a postoperative stay of 10 days.

The patient’s recovery was uneventful. A postoperative echocardiograph indicated that no myxomas remained in any of the cardiac chambers. Follow-ups were performed at 3, 6, and 33 months after surgery. In each follow-up, echocardiography indicated no myxomas. The patient was evaluated as being in New York Heart Association functional class I.

**Figure 5:**
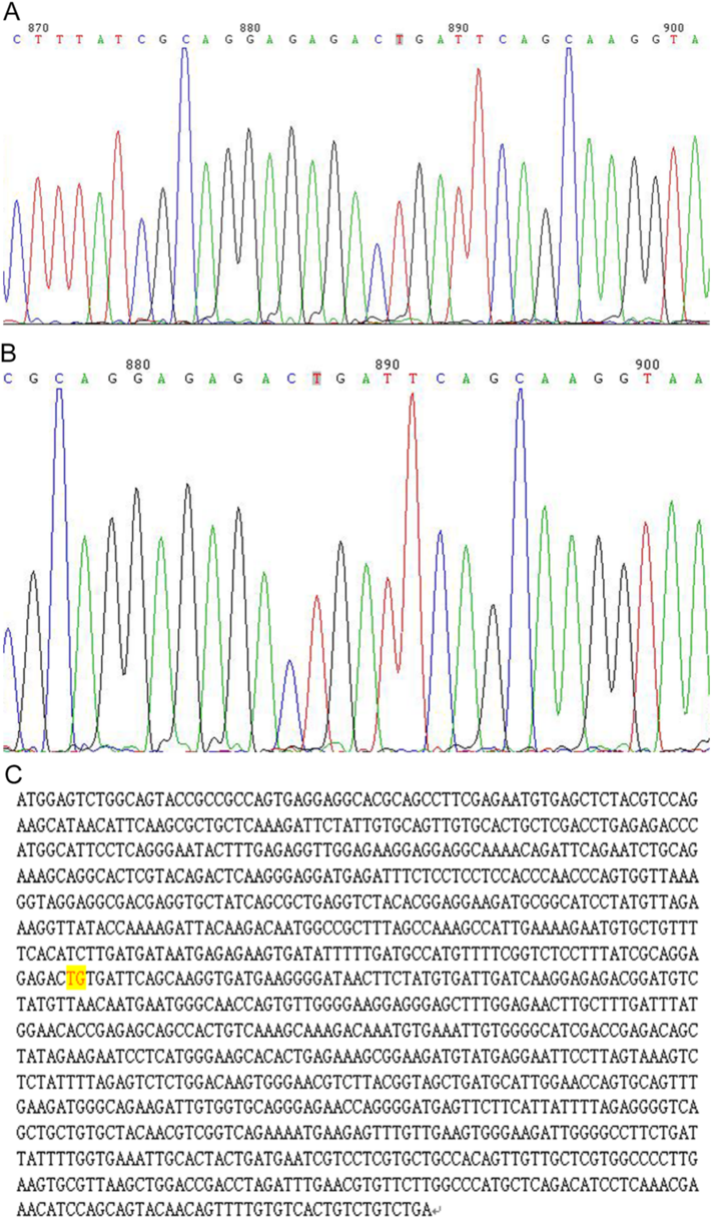
**Genomic sequencing from patient 1 and patient 2.** Genomic DNA was sequenced from both patients for mutations in the PRKAR1A gene: **(A)** patient 1 - c.491_492delTG; **(B)** patient 2 - c.491_492delTG; and **(C)** normal control.

Beijing Ginkgo Wheat Biotechnology Co., Ltd, Beijing, 100084, China, provided genomic sequencing of the patient’s DNA in the PRKAR1A gene. The results indicated the presence of a c.491_492delTG mutation (Figure 5A). The genomic DNA was isolated from the peripheral blood lymphocytes and amplified by PCR using standard methods with the primers listed in Table 2. The gel purified PCR products were then sequenced in both directions on an automated sequencer (ABI 3700; GinkgoWheat Co. Ltd, Beijing, China). Nucleotides were numbered in accordance with the reference sequence for PRKAR1A (GenBank accession no. NG_007093.3).

**Table 2:** **Novel PRKAR1A oligonucleotide sequences for mutation detection**

**Primer name**	**Sequence (5′-3′)**	**Size(bp)**
1Exon-S	TTTTATTCCCTAGTCCCCACTTC	443
1Exon-A	AGTCAGATTCCTTTTCTTCCAAAA	
2Exon-S	ATTGTTAATGGAAGAAGAGATTGG	407
2Exon-A	TTGTACAGGATGGATGAAGTTCC	
3+4Exon-S	AATACCATAATGTGGCTTGACATT	643
3+4Exon-A	CCAATACAAAGTGTTCTGTCCATC	
5Exon-S	ATATGTTGCTTGATTTTCTTTCCC	356
5Exon-A	TCTCTGTGTCATAAAAATACTTGGC	
6Exon-S	CACTCTTAACTCATTTAACTCGTCA	471
6Exon-A	CGCTTATTAAAAGAAAGAAATATTT	
7Exon-S	GCAAATGCTAGGAATTGGG	418
7Exon-A	GTCTTTTCAGGGGGAATGAGTA	
8Exon-S	ATCTAAGGGCTATCTGCAGGC	457
8Exon-A	GTCAGGTATTCTTAAAAACTAAAGC	
9Exon-S	TTAGCTTGATACGAAGAACCTCG	431
9Exon-A	TCAAAATCTCACACCTTCCCTCT	
10Exon-S	GGAAGGTGTGAGATTTTGATCTT	464
10Exon-A	TGCAATAAAAGCAACTTTCAATATA	

### Case report 2

The patient reported on above has a 22-year-old brother who was also diagnosed with multiple cardiac myxomas in February 2011 and who had undergone surgical treatment in another hospital, the Central Hospital of Handan, Hebei province. Upon physical examination, patient 2 also had spotty skin pigmentation on his face (Figure 3B). Primary symptoms were palpitations and shortness of breath after activity over the previous 2 years that had become aggravated over 15 days by sudden syncope.

Ultrasound examination results showed a mural mass with medium echo (5.7 × 3.4 cm) in the left atrium, and the base of the mass was at the lower interatrial septum near the origin of the anterior mitral valve leaflet; a mural mass with medium echo (7.5 × 5.1 cm) was found in the left ventricle, and the base of the mass was wide and located at the lower ventricular anterior wall; a mural mass with medium echo (3.4 × 2.5 cm) was found in the right atrium, and the base of the mass was relatively wide and located at the trabecular of the right ventricular anterior wall; and a mural mass with medium echo (2.2 × 1.4 cm) was found at the right ventricular outflow tract, and the base was at the anterior wall. The results of further evaluations are presented in Table 1.

Patient 2 underwent surgery on 24 February 2011. Two myxomas were removed from the right ventricle through the tricuspid valve; a 20 × 25 × 30 mm^3^ myxoma from the inlet area and a 30 × 30 × 30 mm^3^ myxoma from the outlet area. In the left atrium, one myxoma measuring 30 × 35 × 40 mm^3^ was attached to the fossa ovalis and removed through an interatrial septum incision. In the left ventricle, a 25 × 30 × 30 mm^3^ myxoma was attached to the apical area and removed through an interatrial septum incision and the mitral valve. The cardiopulmonary bypass time was 105 min, and the aortic cross-clamp time was 68 min.

Follow-up on patient 2 was initially performed 3 months after surgery. Echocardiography revealed a 10 × 15 × 20 mm^3^ myxoma remaining in the left ventricle (Figure 1C). At 46-month follow-up, the size of the myxoma in his left ventricle was considered to be stable, and the estimated size this time was 9 × 15 × 21 mm^3^.

Beijing Ginkgo Wheat Biotechnology Co., Ltd, Beijing, 100084, China, provided genomic sequencing of the patient’s DNA in the PRKAR1A gene. The results indicated that he shared the same c.491_492delTG mutation, as his sibling patient 1 (Figure 5B).

## Conclusions

There are two diagnostic guidelines for Carney complex. In the first, at least two major criteria need to be present for diagnosis. In the second, one major clinical manifestation and the presence of one supplemental criterion is sufficient for diagnosis [2] (Table 3). Patient 1 and his brother had multiple cardiac myxomas, spotty skin pigmentation, PRKAR1A gene mutation, and an affected first-degree relative. Therefore, they meet the established diagnostic guidelines and were diagnosed with Carney complex.

**Table 3:** **Diagnostic criteria for Carney complex**

	**Diagnostic criteria**
Major criteria	
	Skin pigmentation disorders
	1. Spotty skin pigmentation with a typical distribution (lips, conjunctiva and inner or outer canthi, vaginal, and penile mucosa)
	2. Blue nevus, epithelioid blue nevus (multiple)
	Myxomas
	1. Myxoma (cutaneous and mucosal)
	2. Cardiac myxoma
	3. Breast myxomatosis or fat-suppressed magnetic resonance imaging findings suggestive of this diagnosis
	4. Osteochondromyxoma
	Endocrine tumors/overactivity
	1. PPNAD or paradoxical positive response of urinary glucocorticosteroids to dexamethasone administration during Liddle’s test
	2. Acromegaly due to GH-producing adenoma
	3. LCCSCT or characteristic calcification on testicular ultrasonography
	4. Thyroid carcinoma or multiple, hypoechoic nodules on thyroid ultrasonography, in a young patient
	5. Psammomatous melanotic schwannoma
	6. Breast ductal adenoma (multiple)
Supplemental criteria	
	1. Affected first-degree relative
	2. Inactivating mutation of the PRKAR1A gene

The case studies presented here suggest to us these important points: Myxoma in patients with Carney complex always has an early onset, but we should be careful if the patient with multiple myxoma is under 40 years old. The following assessments should be undertaken: skin exam, ask for family history, or PRKAR1A gene test if necessary. The recurrence of myxoma is quite common after surgery. But in case 2, the recurrence occurred early, within 3 months after surgery, and the size of myxoma did not grow with a quite long follow-up, while case 1 did not have any recurrence. This may suggest that the most important period is within 3 months of surgery.

In general, cardiac myxomas occur sporadically as an isolated left atrial mass attached to the fossa ovalis. They generally occur between the third and sixth decades of life, and most do not recur after surgical resection. However, Carney complex cardiac myxomas occur in families and usually at considerably younger ages than non-familial myxomas. Single myxoma associated with Carney complex can occur, but most often there are more than one and they occur in any cardiac chamber. Following surgical resection, Carney complex myxomas frequently recur both at the initial resection site and at distant sites [1]. Both patients had multiple cardiac myxomas. Patient 2 had a recurring myxoma in the left ventricle 3 months after the operation. Unlike most myxomas, in these cases, the myxomas were attached directly to cardiac muscle and did not have pedicels.

Carney complex is most commonly due to mutations in the PRKAR1A gene on chromosome 17q22-q24. This gene may function as a tumor-suppressor gene encoding the regulatory subunit type 1-alpha (R1) of protein kinase A. Recently, mutations in the PRKAR1A gene were estimated to occur in more than 60% of Carney complex patients [3]. Approximately 20% of the families affected with Carney complex have 2p16 mutations. To date, there are at least 117 pathogenic variants in PRKAR1A that have been identified [4-7]. In this study, we identified a PRKAR1A mutation, c.491_492delTG, in both patients.

## Consent

The study protocol was approved by the Ethics Committees of Fuwai Hospital, Beijing, and all of the parents provided written informed consent.

## References

[1] Horvath A, Bertherat J, Groussin L, Guillaud-Bataille M, Tsang K, Cazabat L (2010). Mutations and polymorphisms in the gene encoding regulatory subunit type 1-alpha of protein kinase A (PRKAR1A): an update. Hum Mutat..

[2] Stratakis CA, Kirschner LS, Carney JA (2001). Clinical and molecular features of the Carney complex: diagnostic criteria and recommendations for patient evaluation. J Clin Endocrinol Metab..

[3] Sandrini F, Stratakis C (2003). Clinical and molecular genetics of Carney complex. Mol Genet Metab..

[4] Rothenbuhler A, Stratakis CA (2010). Clinical and molecular genetics of Carney complex. Best Pract Res Clin Endocrinol Metab..

[5] Bertherat J (2006). Carney complex (CNC). Orphanet J Rare Dis..

[6] Vezzosi D, Vignaux O, Dupin N, Bertherat J (2010). Carney complex: clinical and genetic 2010 update. Ann Endocrinol (Paris)..

[7] Halaszlaki C, Takacs I, Butz H, Patocs A, Lakatos P (2012). Novel genetic mutation in the background of Carney complex. Pathol Oncol Res..

